# User-Centered Development of Bolster, an mHealth Intervention for Early Psychosis Caregivers: Needs Assessment, Prototyping, and Field Trial

**DOI:** 10.2196/50522

**Published:** 2023-11-30

**Authors:** Benjamin Buck, Mary Wingerson, Erica Whiting, Jaime Snyder, Maria Monroe-DeVita, Dror Ben-Zeev

**Affiliations:** 1 Behavioral Research in Technology and Engineering (BRiTE) Center Department of Psychiatry and Behavioral Sciences University of Washington Seattle, WA United States; 2 Information School University of Washington Seattle, WA United States; 3 Supporting Psychosis Innovation through Research Implementation and Training (SPIRIT) Lab Department of Psychiatry and Behavioral Sciences University of Washington Seattle, WA United States

**Keywords:** caregiving, psychosis, mobile health, mHealth, user-centered design, mobile phone, artificial intelligence, AI

## Abstract

**Background:**

Caregivers play a critical role in the treatment and recovery of youth and young adults at risk for psychosis. Caregivers often report feeling isolated, overwhelmed, and lacking in resources. Mobile health (mHealth) has the potential to provide scalable, accessible, and in-the-moment support to caregivers. To date, few if any mHealth resources have been developed specifically for this population.

**Objective:**

The aim of this study was to conduct user-centered design and testing of an mHealth intervention to support early psychosis caregivers.

**Methods:**

We conducted a multiphase user-centered development process to develop the Bolster mobile app. In phase 1, a total of 21 caregivers were recruited to participate in a qualitative needs assessment and respond to an initial prototype of the Bolster platform. Content analysis was used to identify key needs and design objectives, which guided the development of the Bolster mobile app. In phase 2, a total of 11 caregivers were recruited to participate in a 1-week field trial wherein they provided qualitative and quantitative feedback regarding the usability and acceptability of Bolster; in addition, they provided baseline and posttest assessments of the measures of distress, illness appraisals, and family communication.

**Results:**

In phase 1, participants identified psychoeducation, communication coaching, a guide to seeking services, and support for coping as areas to address. Live prototype interaction sessions led to multiple design objectives, including ensuring that messages from the platform were actionable and tailored to the caregiver experience, delivering messages in multiple modalities (eg, video and text), and eliminating a messaging-style interface. These conclusions were used to develop the final version of Bolster tested in the field trial. In phase 2, of the 11 caregivers, 10 (91%) reported that they would use Bolster if they had access to it and would recommend it to another caregiver. They also reported marked changes in their appraisals of illness (Cohen *d*=0.55-0.68), distress (Cohen *d*=1.77), and expressed emotion (Cohen *d*=0.52).

**Conclusions:**

To our knowledge, this study is the first to design an mHealth intervention specifically for early psychosis caregivers. Preliminary data suggest that Bolster is usable, acceptable, and promising to improve key targets and outcomes. A future fully powered clinical trial will help determine whether mHealth can reduce caregiver burdens and increase engagement in services among individuals affected by psychosis.

## Introduction

### Background

Caregivers, or individuals who provide ongoing support or help to an individual (usually a family member, eg, a child, spouse, or other relative) with a mental illness [1], play a critical role in the recovery of young adults at risk for psychosis [2]. Most youth with early psychosis live with a caregiver [3], and these caregivers are often the first to detect and respond to the signs of psychosis in their loved ones [4]. They also often seek resources and treatment [5], establish contact with providers [6], or work to persuade the individual to make such contacts on their own [7]. Furthermore, once their loved ones are connected to care, caregivers provide logistical and emotional support [8]. Having a caregiver engaged in supporting care is associated with increased engagement in services [9,10] and improved outcomes [11,12].

Caregivers face significant barriers and challenges in their efforts to facilitate help seeking. Many lack accurate information about psychosis and are prone to misattribute symptoms to substances or developmental transitions [13]. Stigmatizing attitudes related to mental illness and help seeking can manifest in unwillingness to reach out to providers or disbelief in the seriousness of symptoms [14]. Caregivers are also prone to severe levels of distress related to confusion, frustration, and worry induced by responding to a loved one’s psychotic episodes [15] and the experience of mental illnesses of their own [16,17]. Better caregiver knowledge of psychosis symptoms is linked with an increased likelihood of recommending professional help seeking for the affected person [18]. However, caregivers commonly report a skill deficit when faced with the challenges of caregiving in the context of psychosis. They often report feeling isolated [19], distressed, and unable to help [20]. Without effective caregiving skills, they are vulnerable to a communication style that is overly emotionally involved, alienating, or hostile (ie, interactions high in *expressed emotion*) [21,22]. This interaction style can worsen family communication [23], which impedes the process of treatment facilitation [24] and is linked with elevated symptoms [25-27] and increased risk for future episodes [28-32]. Family psychoeducation interventions are designed specifically to address these needs; however, specialty psychosis clinics that offer them are out of reach for families who—as a result of limited access or reluctance—have not yet accessed them or are unable to engage in services (eg, because of the distance from clinics and other responsibilities). To address these needs, caregivers often search the internet for resources, information, and support early in the help-seeking process [33]. A majority of caregivers report dissatisfaction with the illness-related information available to them, and they report particular difficulties accessing necessary and actionable information when it is most needed [34]. Caregivers report that although information can be accessed easily on the internet, much of it lacks direct and actionable steps [4].

Mobile health (mHealth) may provide unique advantages in addressing the needs of early psychosis caregivers. First, mHealth tools can serve as *just-in-time* interventions [35], wherein users can use content that responds to particular in-the-moment needs. Caregiving challenges can unfold in an unpredictable manner; optimal resources for caregivers may be those that are available in the environments and moments when these needs emerge. mHealth could also enable more frequent ongoing engagement with intervention content than is possible through psychoeducational websites, many of which provide information in a single large bolus. Second, mobile apps have greater penetration than other web-based platforms. In a day’s engagement with media, the average adult spends much more time engaged with mobile devices than internet-connected computers [36]. Third, mHealth interventions show promise in their scalability. A new generation of self-guided interventions have demonstrated efficacy in a number of psychiatric concerns, including depression [37], anxiety [38], and psychosis [39]. Such interventions can be provided rapidly, at low cost, to individuals who face significant barriers to access the traditional modes of mental health services.

### mHealth Interventions for Caregivers

mHealth interventions designed for caregivers lag behind those designed for individuals with psychosis or other mental health conditions. A recent systematic review of digital technologies for early psychosis caregivers examined 8538 studies and identified no mHealth intervention designed for this high-need population [40]. Given the fact that many mental health care settings focus exclusively on the needs of the identified patient and lack specific services for caregivers [1], self-guided digital tools might fill a particular glaring need in this population. There is a need for literature describing the specific applications of mHealth to address the needs of caregivers of individuals at risk for psychosis. Our team has conducted a multiphase user-centered design and development process to develop an mHealth intervention for caregivers of individuals with early psychosis to support treatment facilitation, including a qualitative needs assessment, live prototyping sessions, and a field trial. The finished product—Bolster—is one of the first ever mHealth interventions designed specifically to support caregivers of young adults with early psychosis. In this paper, we describe the user-centered design process through which Bolster evolved from a set of design objectives to a prototype to a fully functional mHealth support tool.

## Methods

### Overview

Following examples from our team [41,42] and others [43,44], we conducted a multiphase user-centered design and development process that aimed to optimize the Bolster intervention to meet the needs of caregivers of young adults at risk for psychosis. This user-centered development process involved 2 phases. In study phase 1, a total of 21 caregivers participated in a qualitative needs assessment to identify key intervention goals and engaged in a live interaction with a preliminary prototype. Our team analyzed the results of this first phase to develop and optimize the beta version of the Bolster intervention. In study phase 2, a second sample of 11 caregivers participated in a 1-week field trial of the Bolster mobile app.

### Recruitment

Several channels were used to recruit participants. First, advertisements were purchased on Google by the research team to target individuals using particular search terms (eg, “schizophrenia symptoms,” “psychosis,” and “bipolar symptoms”) and iteratively optimized using the Google Ads *broad match* algorithm. Second, the study team placed advertisements in the newsletters of multiple advocacy organizations (eg, the National Alliance on Mental Illness [NAMI] and Mental Health America) and sent email postings to national networks focused on early psychosis (eg, the Psychosis-Risk and Early Psychosis Program Network [PEPPNET] and Washington state’s New Journeys network) encouraging distribution to caregivers served by members of these networks. In each of these postings or flyers, participants were linked to a study landing page that summarized the study and contained links to the consent form and eligibility survey. After opting to take the eligibility survey, participants were required to read the study consent form and confirm understanding in comprehension questions covering study key points. All participants were contacted by a member of the study team to confirm and inform participants of their eligibility status.

### Participants

Across study phases, participants were 32 caregivers of youth and young adults who had experienced symptoms consistent with psychosis. All met the following inclusion criteria: (1) responses of *somewhat agree* or *definitely agree* to ≥2 items on the Caregiver Prime Screen–Revised (CGPS-R) [45]; (2) loved one met the criteria of psychosis onset (per caregiver report) following definitions derived from previous work [46,47], including (2A) experiencing at least 1 positive symptom of psychosis and (2B) at least 2 of the following: serious deterioration of functioning, marked social withdrawal, persistent self-neglect, or episodic marked anxiety; and (3) within the past 5 years, caregivers became aware of their loved one’s symptoms, their loved one first experienced a psychotic episode, or their loved one first engaged in treatment for psychosis. To represent varying caregiver experiences concerning treatment engagement, the first sample (ie, those completing the needs assessment and prototyping) was stratified such that only half provided caregiving to an individual who was already established in specialty services for psychosis. In the phase 2 field trial, all participants were caregivers of young adults who were engaged in specialty psychosis programs.

### Phase 1: Intervention Development

#### Procedures

In the study’s first phase, we aimed to develop Bolster through identifying user needs and gathering concrete feedback in response to a preliminary prototype. This study builds on our team’s previous quantitative work [33] by gathering detailed qualitative data on needs and preferences as well as more direct and actionable information in response to a concrete prototype. Participants completed semistructured needs assessment interviews followed by live prototype interaction sessions through videoconferencing links provided by the research team. The interviewer (MW) was a BA-level research coordinator following a semistructured interview guide developed by senior team members with experience in user-centered design; these senior team members also supervised the interviewer. The interviewer had no established previous relationship with participants, and she provided them with the goals of the research project during the informed consent process. Participants used their own devices for videoconferencing and thus could choose their own environments to complete data collection. No other individuals were present for these interviews aside from the interviewer and the participant. Qualitative interview topics included barriers and facilitators of help seeking, currently available resources that support caregiving, unmet needs in supporting the affected relative’s help seeking, and proposed digital solutions to better meet these needs (ie, what ideal digital supports the participant would create to meet their needs if they had a *magic wand*). Live prototyping sessions involved engaging with a preliminary but fully responsive prototype. This prototype included the following features: an interactive messaging-style (ie, preprogrammed rule-based *chatbot*) interface where users interacted with the system as it provided psychoeducational content. Example modules focused on caregiver resilience (ie, the importance of taking care of oneself to more effectively provide caregiving support) and the communication skill of using *I-statements*. Additional modules involved a video-based deep breathing exercise and a text-based psychoeducation page introducing the stress-vulnerability model of psychosis. This prototype allowed the demonstration and testing of the structures and functions of the platform as well as sample content to allow test participants to provide feedback. The Bolster prototype was connected to the videoconferencing interview session, and cursor control was provided to the participants such that they could click through and respond to prompts in the app remotely. Participants were asked to complete *tasks* (eg, complete a module) while *thinking aloud*. In this approach, participants are asked to express reactions to the functions and content of the app as they come to mind and after completing each task. Interview sessions lasted approximately 90 minutes and were audio recorded and later transcribed for research team analysis. Participants were compensated with US $75 gift cards for their time. There were no additional contacts with participants for repeat interviews or feedback on findings.

#### Analysis

Participants’ qualitative responses were analyzed for key themes using conventional content analysis [48]. Interviews were segmented such that themes could only be coded in relevant sections of the interview. Two coders independently assigned first-level codes (concise summaries no longer than a phrase, eg, “Ability to persuade loved one to take her medication”) to segments of participants’ responses. The 2 coders grouped first-level codes and collaboratively developed second-level interpretive codes (eg, “Communication with loved one”) that also serve as themes reported here. Second-level codes were developed iteratively following discussion of first-level coding and compiled into a codebook with thorough definitions and rules for application in text. The coders then independently returned to the text to apply second-level codes to interview segments. A high degree of agreement was established between the coders (κ>0.72), and all disagreements were resolved through consensus discussion. Prototype reactions (ie, problematic or positive interactions and issues noted in the *think aloud* sessions) were listed exhaustively to provide a comprehensive review of areas to adjust in the final version of Bolster. We also report on our team’s design objectives and overall features of the version of Bolster tested in the phase 2 field trial.

### Phase 2: Field Trial

#### Procedures

This field trial deployed the updated version of Bolster based on phase 1 feedback in a sample of caregivers for 1 week. Eligible participants were texted links to a battery of baseline questionnaires (refer to the next subsection). Once these questionnaires were completed, a study team member scheduled and completed a remote *installation session* wherein the study team member ensured that Bolster was installed on the participants’ own device and provided orientation to the app, the 1-week testing period, and the remaining assessment schedule. Participants were encouraged to use the app as they would normally but also to do so in a manner that allowed them to provide honest and detailed feedback. If participants did not engage in the app for multiple consecutive days, a member of the study team contacted the participant on the third day to ensure that the platform was functioning properly or to provide a reminder of the upcoming end of the testing period. This type of outreach occurred for 4 (36%) of the 11 participants. Participants were sent links to complete the full baseline assessment battery a second time on day 8. Following completion of the postintervention questionnaires, participants were invited to postintervention qualitative interviews. In these interview sessions, participants connected to the videoconferencing platform with their own smartphones such that the interviewer could observe their interactions with the platform in real time. During these interviews, participants were asked (1) to provide ratings of (1A) how easy to use and (1B) useful they found each feature, as well as (2) to show on their devices something memorable and representative (positive or negative) of their experience with Bolster. Participants were compensated with a US $50 gift card per completed assessment (i.e. baseline and post-test) as well as a US $40 gift card for completing a qualitative interview.

#### Measures

Bolster was designed to have an impact on the primary targets of illness knowledge, illness appraisals (caregiving-related appraisals and psychosis-related appraisals), and coping in hopes that changes in these primary targets would result in improvements in the primary outcomes of caregiver distress and expressed emotion. Illness knowledge was assessed with the Knowledge About Schizophrenia Test (KAST) [49], an 18-item multiple-choice assessment that scored participants’ knowledge of the etiology, symptoms, and prognosis of schizophrenia on the number of items answered correctly. The Illness Perception Questionnaire for Schizophrenia: Relatives’ version (IPQSR) [50] is a self-report scale of caregivers’ beliefs about the severity, prognosis, and responsiveness to treatment of mental illnesses and used to assess caregiver knowledge of illness appraisals. We administered subscales focused on emotional representation (high scores reflecting high levels of distress related to the condition), incoherence (high scores reflecting lacking an understanding of the illness), and the measures of control (high scores reflecting belief that the actions of the individual and the caregiver can affect the course of the illness) and consequences (high scores reflecting a perception that psychosis results in greater negative consequences for the individual and the caregiver; given evidence from Lobban et al [50] suggesting relationships between the caregiver version and patient version of each *consequences* and *control* subscale, these were combined for simplicity). Caregiving-related appraisals were assessed with the Experiences of Caregiving Inventory (ECI) [51], a 66-item assessment of the perceived impact of caregiving on the individual’s life, with subscale scores for both negative and positive experiences. Coping was assessed with 2 measures: the Brief Coping Orientation to Problems Experienced Inventory (Brief-COPE) [52], a 28-item self-report scale of coping skills in response to stressors, with 16 items assessing coping skills proposed a priori as positive [51]; and the Coping Self-Efficacy Scale (CSES) [53], a 26-item self-report questionnaire measuring the perceived ability to cope with various life challenges. Expressed emotion was assessed using the Family Questionnaire (FQ) [54], a 20-item self-report assessment of emotional expression in family members toward patients with mental illness, and caregiver distress was assessed a revised version of the General Health Questionnaire, 12-item version (GHQ-12) [55,56], a self-administered questionnaire that measures general psychological distress. The scale was revised consistent with recent psychometric studies examining the confounds of wording effects on this measure [57] in that scale items were kept consistent (ie, ranging from *not at all* to *much more than usual*). Positively worded items are reverse scored such that higher scores indicate greater distress.

#### Analysis

Analyses were conducted using SPSS software (version 28.0; IBM Corp). Acceptability and usability were assessed through a review of individual items on the modified System Usability Scale. With regard to use statistics, we also reviewed the following metrics: (1) the percentage of days on which participants opened Bolster of those days on which they had access, (2) the number of minutes per day that participants used Bolster, and (3) the number and rate of completion of Bolster lessons and practices. We conducted exploratory analyses of clinical outcomes using paired sample 2-tailed *t* tests, assessing significant within-individual change during the testing period and reviewing effect sizes.

### Ethical Considerations

The institutional review board of the University of Washington approved all study procedures (STUDY00013334). All participants provided informed consent to participate. Any identifiable information was kept on secure and password-protected servers; data were deidentified for analysis.

## Results

### Participant Characteristics

Participant demographics across study phases are provided in Table 1. The sample consisted almost exclusively of women (31/32, 97%) and parents (31/32, 97%) aged approximately 50 to 55 (mean 50.19, SD 10.74 in phase 1; mean 55.27, SD 7.85 in phase 2) years. The affected relative was on average aged in their early to mid-20s and had been experiencing symptoms of psychosis for approximately 3 years. The majority of participants (23/32, 72%) were non-Hispanic White. Most of the caregivers (22/32, 69%) endorsed a diagnosis of schizophrenia for their loved one and endorsed symptoms and scores on the CGPS-R that reflected a high severity of illness. As stipulated by our a priori stratified recruitment strategy in phase 1, a total of 10 (48%) of the 21 participants were caring for individuals who were not engaged in treatment or were in the process of supporting their loved one seeking a higher level of care. The remainder (11/21, 52%) were caring for individuals who were already established in specialty mental health services. Across phases, of participants whose loved ones were engaged in specialty mental health services, the majority (17/22, 77%) were engaged in coordinated specialty care programs. No participant dropped out or refused to participate in any study procedures.

**Table 1:** Demographic characteristics of study participants.

Characteristics			Sample 1: needs assessment and prototyping (n=21)		Sample 2: field trial (n=11)
Age (y), mean (SD)			50.19 (10.74)		55.27 (7.85)
Age (y) of loved one, mean (SD)			23.33 (5.18)		24.55 (3.36)
**Sex, n (%)**					
	Female	20 (95)		11 (100)	
	Male	1 (5)		0 (0)	
**Gender, n (%)**					
	Woman	20 (95)		11 (100)	
	Man	1 (5)		0 (0)	
**Relationship to loved one, n (%)**					
	Parent	20 (95)		11 (100)	
	Sibling	1 (5)		0 (0)	
**Race and ethnicity, n (%)**					
	Asian	3 (14)		1 (9)	
	Black or African American	1 (5)		2 (18)	
	Hispanic White	2 (10)		0 (0)	
	Non-Hispanic White	15 (71)		8 (73)	
**Loved one’s diagnosis^a^, n (%)**					
	Schizophrenia or schizoaffective disorder	14 (67)		8 (73)	
	Bipolar disorder	8 (38)		6 (55)	
	PTSD^b^	5 (24)		3 (27)	
	Major depressive disorder	6 (29)		2 (18)	
	Other psychotic disorder	4 (19)		1 (9)	
	Not sure or prefer not to say	1 (5)		1 (9)	
CGPS-R^c^, mean (SD)			51.33 (9.88)		54.88 (10.10)
CGPS-R (number of items rated *somewhat agree* or higher), mean (SD)			6.33 (2.33)		7.09 (2.98)
**Symptoms endorsed, n (%)**					
	Hallucinations	17 (81)		10 (91)	
	Thought disorder	19 (91)		11 (100)	
	Delusions	20 (95)		10 (91)	
	Bizarre or erratic behavior	20 (95)		10 (91)	
	Strange psychomotor behavior	11 (52)		7 (64)	
**Functional impacts endorsed, n (%)**					
	Deterioration in daily functioning	19 (91)		11 (100)	
	Social withdrawal	18 (86)		11 (100)	
	Persistent self-neglect	13 (62)		7 (64)	
	Severe anxiety or agitation	20 (95)		10 (91)	
Years since first aware of symptoms, mean (SD)			2.93 (2.15)		3.63 (2.69)
Years since first episode (if applicable), mean (SD)			2.98 (2.07)		3.01 (2.34)

^a^Coded nonexclusively.

^b^PTSD: posttraumatic stress disorder.

^c^CGPS-R: Caregiver Prime Screen–Revised.

### Phase 1: Intervention Development

#### Qualitative Needs Assessment

During the course of the analysis, the study team observed that the structure of 2 qualitative interview prompts—unmet needs and ideal supports (the *magic wand* question)—often generated responses that aligned with one another or reiterated key themes (eg, unmet need of *actionable information related to help seeking* and ideal feature of *an actionable guide to help seeking*). In light of this, our team analyzed and interpreted these items together (refer to Table 2 for examples and theme frequencies). Thus, these themes reflected salient areas to address in a digital intervention based on the ways in which they represented current unmet needs. Themes fell into four broad categories: (1) communication coaching, (2) general psychoeducation, (3) guide to treatment seeking, and (4) support for caregiver coping.

**Table 2:** Combination of themes from unmet needs and magic wand items linked to Bolster elements (n=21).

Category and theme identified in response to *unmet needs* prompt		Theme frequency, n (%)	Theme identified in response to *magic wand* prompt		Theme frequency, n (%)		Bolster elements (ie, ways to address themes using scalable self-guided mHealth^a^)
**Communication coaching**							
	Communication with loved one	18 (86)	Tool supporting communication with loved one	11 (52)		Interactive communication coaching such that the caregiver can practice various communication skills in the app via text or be coached through how to complete an in vivo practice session	
	Communication with others about psychosis	7 (33)	No theme identified	—^b^		Lessons and practices that target stigmatizing attitudes alongside information on strategies for how to talk with others about your loved one’s experience	
**Psychoeducation**							
	General information and knowledge of psychosis	11 (52)	Psychosis-related information and guide to recognizing symptoms Expert Q&A^c^	11 (52) 5 (24)		*Psychosis 101* lessons providing introduction to key points about psychosis	
	General information and knowledge of treatments and medications	7 (33)	Treatment-related information	9 (43)		*Psychosis 101* lessons providing introduction to key points about treatment and recovery	
	No theme identified	—	Tracking loved one’s symptom presentation	6 (29)		Structured instrument to aid caregivers in tracking symptom changes over time	
**Guide to seeking services**							
	Actionable guide to help seeking	11 (52)	Actionable guide to help seeking	11 (52)		Actionable step-by-step guide on how to encourage your loved one to seek services, as well as how to go about finding appropriate treatment for your loved one	
	Actionable guide to other (ie, nonmedical) resources	5 (24)	Actionable guide to nonmedical or nonpsychiatric resources	7 (33)		Links to information about resources related to housing, employment, or food security	
	Planning for, and responding to, emergencies and crisis situations	7 (33)	Crisis-related information and support	9 (43)		Step-by-step guide to evaluating and responding to a crisis, as well as links to emergency resources if caregiver is responding to crisis in the moment	
**Support for coping**							
	Support for caregiver’s coping	11 (52)	Peer connections Self-guided support for caregiver well-being	9 (43) 8 (38)		Video and interactive text-based practices supporting cognitive restructuring (eg, self-compassion), behavioral exercises (eg, heathy habits), and simple mindfulness practices (eg, deep breathing)	

^a^mHealth: mobile health.

^b^Not applicable.

^c^Q&A: question and answer.

Responses related to communication were common and salient in many interviews, at times regarding communication with others (eg, family and friends) about the loved one’s symptoms but primarily in relation to communicating directly with the loved one and especially in response to difficult topics (eg, in response to delusions, disorganization, or opposition to engagement in mental health treatment):

How can you give her support when it doesn’t make sense because the support she wants doesn’t make any sense because it’s not real?...Learning how to support them without trying to make them think that you believe what they’re saying is true.

Unmet needs, participant 67

So, if there was a nice app that said “Okay, people in psychosis may interact with you like this and here are some techniques to respond and here’s when you need to disengage,” because it was clear to me at some points that the best thing I could do was get out, my presence was not helpful.

Magic wand, participant 5

On the basis of insights from caregivers related to this topic, the project team ensured that Bolster was primarily oriented around communication skills, including both foundational listening skills (eg, active listening) and those related to particular challenging topics (eg, responding to delusions). This content would provide both clear directives and opportunities to practice new skills.

Caregivers also commonly reported feeling overwhelmed and in need of support in coping with this demanding role. This manifested in desires for reminders to engage in self-care and supportive connections with peer caregivers who had shared experiences:

How do you take care of yourself, remembering to take care of yourself. Because you’re so much into the problem, that then you forget you. So people forget themselves to the extent that they start failing in their jobs, and that’s important.

Unmet needs, participant 126

[T]o have a trained person who can work with you on developing boundaries and helping you create a strategy, helping to hold you accountable to the things you said you were going to do. That would be really helpful.

Magic wand, participant 66

Caregivers noted the common experience of neglecting their own needs and well-being, often acknowledging the dissonance between an awareness of the importance of one’s own needs while lamenting the frequency with which they neglected them.

Caregivers emphasized 2 broad categories related to informational needs as well, including general psychoeducation (eg, information about psychosis as well as its treatment and prognosis) and a guide to engaging in the mental health services system, both for ongoing outpatient treatment and in response to psychiatric crises. Many participants noted that although informational resources exist on the web (and in concert with treatment programs), it is not always easy to tell which sources are trustworthy, and even among those that seem to be trustworthy, many provide general overviews more effectively targeting a general or academic audience rather than a caregiver needing direct guidance in response to specific challenging situations:

When you research something like that, you have to read a lot of different sources, so having one place that has as much information as it can pack in, that explains in a way that anyone can understand.

Unmet needs, participant 73

I didn’t know how to find support, I didn’t have a list of potential therapists outside of the [treatment] program...He came home untreated in a lot of ways and I didn’t have any direction on how to find those resources.

Unmet needs, participant 82

In response to the magic wand item, a number of caregivers (6/21, 29%) also reported a desire to have the ability to assess, track, and monitor changes in symptoms over time, either to be able to determine whether symptoms were improving or to more easily report changes to providers at future clinical visits. Two additional prompts in the qualitative interview focused specifically on the positive features of existing resources that caregivers had used for support as well as attributes that were negative or lacking. First, digital or web-based resources that caregivers had found useful were most commonly described as detailed (12/21, 57%), hopeful (11/21, 52%), and providing clear actionable steps for help seeking (4/21, 19%) or in communicating with their loved one (3/21, 14%). Frustrations that were related to web-based resources often stemmed from such resources simply being absent (7/21, 33%) or the fact that existing resources were provided in the context of clinical services that caregivers found to be inadequate or unhelpful (10/21, 48%); were lacking in detail (3/21, 14%); discouraging (5/21, 24%); or, again, lacking clear actionable guidance (7/21, 33%):

I mean there’s a lot of general information about psychosis, Google is there...When you see somebody who’s in psychosis, you don’t need to read an article about what is psychosis. You’re like, “I know what psychosis is, thank you.” But what do you do? General information isn’t helpful when you are in a crisis situation.

Disappointing resources, participant 11

#### Prototyping

Open-ended feedback on the Bolster prototype was overall positive, but it also identified areas for improvement. Participants reported finding the information useful and new and the tone of the content to be encouraging and comforting. Several participants were particularly enthusiastic about the opportunities for interactive practices and reacted positively to opportunities to write their responses into the app, which made the platform feel more personalized.

Most notable among areas for improvement was the messaging-style interface. Participants expressed some frustration with the fact that this method of delivery was not self-paced (eg, “It’s choppy”), and others were confused by whether and from whom app content was being delivered conversationally (eg, “Who am I talking to?”). In addition, many participants expressed a desire for more detailed information in response to the sample psychoeducational prompt, but they also expressed some reticence about providing too much text. One comment that balanced these concerns was a suggestion to provide information in diverse presentation modes (eg, video and audio in addition to text) as well as highlighting key or important points within larger chunks of text. Participants also stressed the importance of setting expectations in advance of each Bolster module (eg, providing a summary statement of the purpose of a module and a time estimate). Several participants reported a desire for a symptom tracker that they could use to track and follow changes in their loved one’s symptoms over time, and, if necessary, share these updates with a provider at a future visit. Most other comments pertained to suggestions for additional content (eg, crisis line telephone numbers and information on communicating with others about psychosis) and minor changes to navigation (eg, adding home, back, and next buttons) or display (eg, greater color contrast and removing particular icons).

#### Insights and Design Objectives

Design objectives were identified based on insights collected from qualitative interviews and prototyping sessions. First, our team identified content areas to develop based on the qualitative needs assessment, including communication coaching, general psychoeducation, information on seeking services, and support for coping. Given the fact that many caregivers expressed concerns about their difficulty *fitting in* additional activities or practices because of the current demands on their time, we resolved to create modules that were brief and simple and that normalized difficulties in making time to engage in the system. In addition, it was important for Bolster content to communicate information with an appropriate tone that balanced accurate information about the seriousness of psychosis with appropriate levity and positive messages about recovery.

Second, participants expressed a desire for clear actionable guidance rather than more general psychoeducational information. On the basis of these comments, a key guiding design principle for Bolster was to provide information and support that was targeted and actionable, as opposed to generic. The Bolster app could provide ongoing scaffolding and support for caregivers that differs from informational websites that are designed to provide overviews of key topics in a few visits (or a single visit). These comments resulted in the proposal of new features, including an index of clear directive guides in responding to common challenging situations (refer to *Action Plans* in the next subsection); curated links to resources, additional information, and treatment listings (refer to *Resources* in the next subsection); and the ability to track the loved one’s symptoms over time (refer to *Tracking* in the next subsection).

Third, participants had difficulties with content delivered in a messaging-style interface. Our team responded to these concerns by altering the method of presentation of psychoeducational content. All message-style interactions were removed. In their place, all psychoeducation lessons were delivered through 2 modalities: videos of a clinician explaining a key idea and a text-based *carousel*. In these carousels, participants could swipe through a series of screens that each introduced 1 key idea in 1 or 2 sentences at a time with a related illustration or icon. This method responds to several key comments made by participants: it is self-paced, highlights key information, and reduces the overall amount of text on each page.

Fourth, given participants’ positive reaction to interactive features (eg, those where they could enter information specific to their situation) and their expressed need for communication skills coaching, more interactive text-based modules were added such that each lesson introducing a skill included an accompanying practice; for example, a video and carousel would introduce the communication skill of using open-ended questions (ie, the *lesson*), and an interactive text-based module (ie, the *practice*) would prompt the user to brainstorm ways to adjust their communication style to include more open-ended questions. The user could choose to engage in either the lesson or the practice on its own or one after the other. Finally, to address concerns about user privacy, our team designed Bolster to only save written text on the user’s device (and not in a database); thus, no one—neither the app developers nor the study team members—could access what the user wrote in free-response items. On the basis of these conclusions, new mock-ups were developed by the research team mapping out design objectives, and these were incorporated by the software developers who built the final source code for Bolster.

#### The Bolster mHealth Intervention

**Figure 1:**
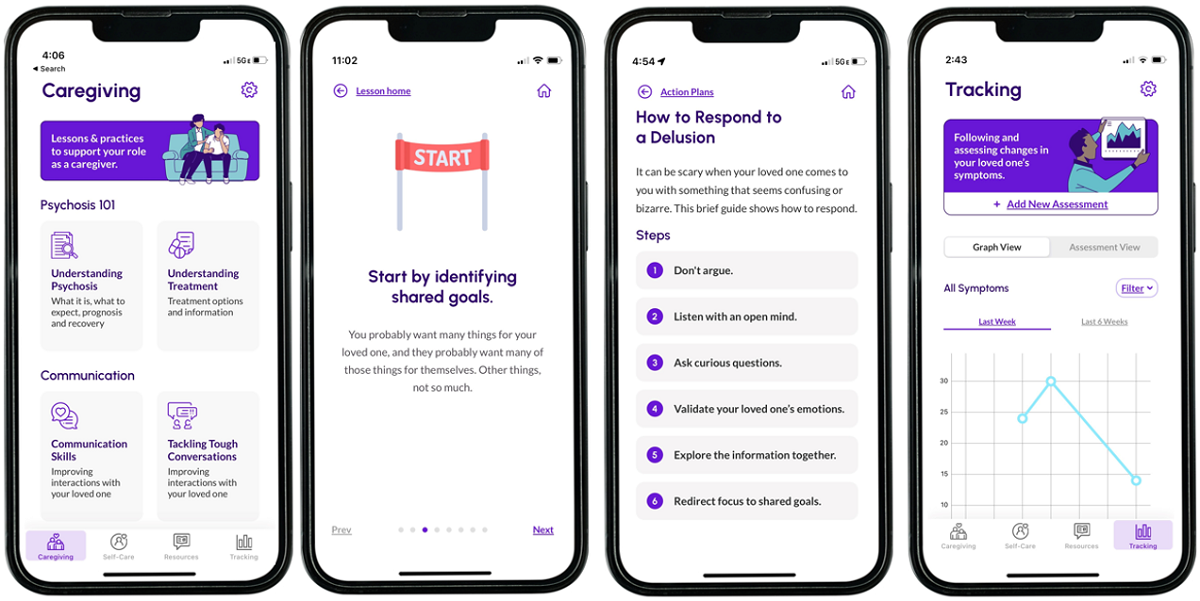
Screenshots of the Bolster final version: (A) the caregiving main menu screen, (B) an example of a lesson page delivered through a swiping carousel, (C) the action plans menu, and (D) the tracking graph view.

The final Bolster mobile app is a web-based mobile app for iOS, with the test version available on the Apple App Store. Intervention content was drafted by the study team, 3 of whom are clinical psychologists with specialized expertise in cognitive behavioral therapy for psychosis and/or family psychoeducation. The Bolster app’s components are based on the cognitive model of caregiving [16], according to which caregiver appraisals lead to emotional and behavioral changes that affect interactions with the affected individual and with service providers. Bolster aims to improve illness and caregiving appraisals and support coping through 4 primary sections (Figure 1). *Caregiving* provides users with psychoeducation and communication coaching through paired lessons and practices. *Self-care* similarly offers paired lessons and practices; however, these focus specifically on skills related to managing one’s own stress and well-being (eg, mindfulness, behavioral activation, and self-compassion). *Resources* offer links to external web pages, treatment listings, and videos of young adults and family members with lived experience describing their experiences. Within the *Resources* tab, the *Action Plans* feature provides users with specific targeted guidance in responding to challenging situations related to caregiving (eg, responding to delusions and encouraging help seeking). Finally, *Tracking* provides users with the ability to enter their perception of their loved ones’ symptoms (based on *Diagnostic and Statistical Manual of Mental Disorders, Fifth Edition* [DSM-5] symptoms of psychosis [58,59]), and then graphs and indexes these ratings so that changes can be tracked over time. Importantly, Bolster was designed such that no identifying information is collected by the app, and nor are written responses stored anywhere in the app or in a database, thus providing users with privacy. Even so, data are encrypted in transit and at rest using secure HTTP and transport layer security.

### Phase 2: Field Trial

#### Use of Bolster

All participants who installed Bolster completed the study, and none were lost to follow-up. Participants opened Bolster on 66% (58/88) of the days that it was available and on average had Bolster open for 26.64 (SD 13.61) minutes per use day. This equated to 14.87 (SD 7.21) minutes per day on average during the testing period overall. Participants opened 22.91 (SD 17.69; 2.86/participant/d) lessons and 10.91 (SD 8.80; 1.32/participant/d) practices on average. Completion rates for practices were quite high: participants fully completed 86.5% (218/252) of the lessons and 80% (96/120) of the practices that they initiated. Among the lessons opened, 47.6% (120/252) pertained to psychoeducation about psychosis and treatment, 29.8% (75/252) to communication skills, and 22.6% (57/252) to self-care skills. Practices were somewhat evenly split between their 2 categories: communication skills (70/120, 58.3%) and self-care (50/120, 41.7%).

#### Usability and Acceptability of Bolster

Participants also reported that Bolster was highly usable. All items on the modified System Usability Scale can be found in Table 3. Of the 11 caregivers, 10 (91%) reported that they would recommend Bolster to another caregiver and that they would use Bolster if they had access to it. Most of the caregivers (9/11, 82%) also reported that they were satisfied with Bolster and would like to use it often. Negatively worded items also consistently reflected positive experiences with Bolster because no participant reported finding Bolster to be inconsistent, awkward, or needing lots of training to use. Nearly all participants (10/11, 91%) disagreed with the item stating that Bolster was very complicated. In postintervention qualitative interviews, participants rated (on scales of usefulness and ease of use ranging from 1 to 10) overall Bolster usefulness on average as 8.95 (SD 0.98) out of 10 and gave an average easy-to-use score of 9.36 (SD 0.50) out of 10. Notably, no participant gave an overall easy-to-use rating of <9 or an overall usefulness score of <7. Qualitative comments typically emphasized how participants enjoyed that Bolster was comprehensive and detailed, that it was simple, had a *clean design*, and was easy to use.

**Table 3:** Participant usability and acceptability ratings (n=11).

Items			Disagree, n (%)		Neutral, n (%)		Agree, n (%)
**Acceptability**							
	“If I have access to Bolster, I will use it”	0 (0)		1 (9)		10 (91)	
	“I would recommend Bolster to another caregiver”	0 (0)		1 (9)		10 (91)	
	“I think that I would like to use Bolster often”	0 (0)		2 (18)		9 (82)	
	“I am satisfied with Bolster”	0 (0)		2 (18)		9 (82)	
	“I feel I need to have Bolster” (n=10)	1 (10)		2 (20)		7 (70)	
	“Bolster is fun to use”	1 (9)		4 (36)		6 (55)	
**Usability**							
	“Overall, I am satisfied with how easy it is to use Bolster”	0 (0)		0 (0)		11 (100)	
	“I felt comfortable using Bolster”	0 (0)		0 (0)		11 (100)	
	“It was easy to learn to use Bolster”	0 (0)		0 (0)		11 (100)	
	“I found that the different parts of Bolster work well together”	0 (0)		1 (9)		10 (91)	
	“I would imagine that most people would learn to use Bolster very quickly”	0 (0)		1 (9)		10 (91)	
	“I felt very confident using Bolster”	0 (0)		1 (9)		10 (91)	
	“I was able to complete the lessons and practices quickly in Bolster”	0 (0)		1 (9)		10 (91)	
	“The information provided for Bolster was easy to understand”	0 (0)		1 (9)		10 (91)	
	“How things appeared on the screen was clear”	0 (0)		1 (9)		10 (91)	
	“I thought Bolster was easy to use”	0 (0)		2 (18)		9 (82)	
	“Bolster helped me with caregiving”	0 (0)		2 (18)		9 (82)	
	“Bolster was interactive enough”	0 (0)		2 (18)		9 (82)	
	“Whenever I made a mistake using Bolster, I could recover easily and quickly”	0 (0)		3 (27)		8 (73)	
	“It was easy to find the information I needed” (n=10)	0 (0)		3 (30)		7 (70)	
	“Bolster works the way I want it to work”	0 (0)		5 (46)		6 (56)	
	“I found Bolster to be very complicated”^a^	10 (91)		0 (0)		1 (6)	
	“I think that I would need the support of a technical person to be able to use Bolster”^a^	10 (91)		1 (9)		0 (0)	
	“I thought there was too much inconsistency in Bolster”^a^	11 (100)		0 (0)		0 (0)	
	“I found Bolster very awkward to use”^a^	11 (100)		0 (0)		0 (0)	
	“I needed to learn a lot of things before I could get going with Bolster”^a^	11 (100)		0 (0)		0 (0)	

^a^Reverse coded such that disagreement denotes higher perceived usability or acceptability.

#### Effects on Targets and Outcomes

An exploratory examination of changes from baseline to posttest assessment (Tables 4 and 5) showed improvements in clinical targets. Participants experienced improvements consistent with medium effects in 3 kinds of illness appraisal or knowledge variables—emotional representation (Cohen *d*=0.63), coherence (Cohen *d*=0.55), and consequences (Cohen *d*=0.68)—as well as in coping self-efficacy (Cohen *d*=0.54). They experienced small improvements in coping skills practiced (Cohen *d*=0.27) and appraisals of caregiving experiences as positive (Cohen *d*=0.26). With regard to primary outcomes, participants experienced large improvements in overall distress (Cohen *d*=1.77) and medium-level improvements in expressed emotion (Cohen *d*=0.52). All assessed outcomes moved in the direction associated with improvement, with the exception of illness knowledge and appraisals related to the *controllability* of psychosis, each of which did not seem to change during the study period. Notably, illness knowledge scores seemed to be affected by ceiling effects because many participants received a high score on the KAST at baseline (mean 16.72 correct out of 20 compared with psychometric work on the initial development of the KAST [49] suggesting typical average scores among family members of 10.9 out of 20 or among lay community members of 9.3 out of 20).

**Table 4:** Baseline and posttest scores of intervention targets.

Variable^a^	Measure	Baseline score, mean (SD)	Posttest score, mean (SD)	2-tailed		
				*t* test (*df*)	*P* value	Cohen *d*^b^
Illness knowledge	KAST^c^	16.72 (1.19)	16.63 (2.01)	−0.28 (10)	.78	−0.09
Illness knowledge	IPQSR^d^: coherence	10.18 (2.64)	8.82 (2.56)	−1.81 (10)	.10	0.55
Illness appraisals	IPQSR: emotional representation	31.90 (5.45)	29.82 (5.91)	−2.10 (10)	.06	0.63
Illness appraisals	IPQSR: consequences	71.72 (8.54)	68.27 (10.26)	−2.03 (10)	.07	0.68
Illness appraisals	IPQSR: control	33.00 (2.76)	32.72 (2.97)	−0.29 (10)	.78	−0.09
Caregiving appraisals	ECI^e^: negative	77.55 (33.72)	75.58 (33.81)	−0.62 (10)	.55	0.19
Caregiving appraisals	ECI: positive	34.90 (9.57)	37.18 (7.47)	0.85 (10)	.42	0.26
Coping	Brief COPE^f^: positive coping	23.97 (9.08)	25.86 (7.20)	0.91 (10)	.39	0.27
Coping	CSES^g^: total	168.18 (59.10)	199.57 (27.77)	1.79 (10)	.10	0.54

^a^Mean imputation (by factor-analytically defined subscale) was used to replace missing values.

^b^Cohen *d* values are scaled such that a positive value denotes movement in the hypothesized direction.

^c^KAST: Knowledge About Schizophrenia Test.

^d^IPQSR: Illness Perception Questionnaire for Schizophrenia: Relatives’ version.

^e^ECI: Experiences of Caregiving Inventory.

^f^Brief COPE: Brief Coping Orientation to Problems Experienced Inventory.

^g^CSES: Coping Self-Efficacy Scale.

**Table 5:** Baseline and posttest scores of intervention outcomes.

Variable^a^	Measure	Baseline score, mean (SD)	Posttest score, mean (SD)	2-tailed		
				*t* test (*df*)	*P* value	Cohen *d*^b^
Distress	GHQ-12^c^	17.48 (3.49)	11.35 (2.34)	−5.88 (*10*)	<.001	1.77
Expressed emotion	FQ^d^	50.22 (11.14)	46.64 (10.92)	−1.72 (*10*)	.12	0.52

^a^Mean imputation (by factor-analytically defined subscale) was used to replace missing values.

^b^Cohen *d* values are scaled such that a positive value denotes movement in the hypothesized direction.

^c^GHQ-12: General Health Questionnaire, 12-item version.

^d^FQ: Family Questionnaire.

## Discussion

### Principal Findings

mHealth could play a useful role in supporting caregivers of young adults at risk for psychosis. mHealth interventions are scalable, acceptable, and can provide in-the-moment support well suited to the challenges of caregiving. Our team developed, optimized, and tested Bolster, an mHealth intervention designed in response to the needs and preferences identified by members of this population. In a qualitative needs assessment, caregivers expressed interest in a tool that provided actionable guidance, opportunities for personalization, and the ability to track changes over time. Prototyping revealed preferences for static text and video (rather than messaging-style interactions), personalization, and interactive features. The intervention that was optimized based on this feedback—Bolster—provides written and video-based lessons and practices focused on psychoeducation, communication skills, and self-care, as well as assessments to track changes in the relative’s symptoms and links to curated resources related to psychosis, treatment, and treatment listings. In a 1-week usability field trial, Bolster seemed promising. Participants found it to be acceptable, usable, and reported marked changes in their appraisals of illness, distress, and expressed emotion. Given the nature of this study as a usability field trial, the lack of a control condition, and the absence of hypotheses about clinical targets and outcomes, the results related to intervention effects are highly preliminary and should be interpreted with caution. However, they do seem to reflect that providing mHealth specific to caregiving support is promising and that additional, larger, controlled studies are warranted.

Our systematic Bolster development process led to several clear conclusions about the needs and preferences of caregivers. First, participant responses suggested needs in a few key areas: psychoeducation, information about seeking services, coping, and communication skills. Second, many caregivers expressed frustration with the lack of *actionable* information available on the internet today and described finding general information about psychosis that did not provide clear guidance in how to respond to caregiving-related challenges. This reflects previous qualitative work in which caregivers expressed the desire for step-by-step guides or instructional resources [4]. Others were disappointed when they found information that they found demoralizing or discouraging about psychosis and its prognosis. Previous literature suggests that caregivers can receive stigmatizing reactions from general practitioners and hospital staff, and the ongoing stigma of psychotic illness persists as a barrier to information and treatment seeking [60,61].

Initial prototype usability testing led to several key principles for the optimization of Bolster. Most notably, the initial messaging-style interface of the prototype seemed to have several limitations, including difficulties in pacing, confusion about the messaging-style interface, and the lack of a mechanism for highlighting key information. It is unclear based on this feedback whether these identify generalizable limitations with messaging-style interventions or chatbots in this population or whether these comments were specific to our prototype. Many other examples of rule-based mental health chatbots have demonstrated promising usability [62,63], and recent and ongoing developments in language-based artificial intelligence technologies [64-66] will likely lead to concurrent innovations that facilitate positive user experiences with these tools. By contrast, many participants reacted positively to interactive text modules wherein they could enter particulars related to their caregiving situation. Such interactive features seem to be linked with improvements in help-seeking behaviors in studies of interventions of this construct [67]. Additional usability changes responded to user comments related to clear expectation setting (eg, time estimates and a summary statement describing modules) and changes to navigation (eg, consistent back, home, and next buttons). The subsequent field trial suggested that the user-centered design process was effective. All participants who completed baseline assessments engaged in the intervention and were retained at 1-week follow-up. Participants provided highly positive usability feedback: nearly all participants (10/11, 91%) reported that they would use Bolster if they had continued access and that they would recommend it to a fellow caregiver. Although exploratory, preliminary analyses of clinical effects unveiled a promising result and suggest that a sufficiently powered trial of Bolster is warranted. Participants provided scores indicating changes in illness knowledge, appraisals, coping self-efficacy, distress, and expressed emotion. Other caregiver-focused interventions, including those delivered in person by providers [68,69] or remotely in asynchronous web-based interactions [70,71], have demonstrated similar promising effects; however, as a fully self-guided mobile intervention, Bolster may have several advantages: it is scalable, can be used repeatedly over long periods of time, and is well suited for in-the-moment needs.

### Limitations

This study has several limitations. First, our user-centered development process was meant to solicit detailed guidance from caregivers with lived experience; study conclusions are drawn from small samples and may not generalize to all members of this population. Furthermore, given the fact that recruitment occurred primarily on the internet, participants may be drawn from a population that is already predisposed to digital tools. Second, design objectives for Bolster were constrained to some extent by predefined project goals. Bolster was a priori proposed as a self-guided mobile intervention. Thus, caregiver feedback related to intervention features that could require clinical support (eg, an expert question-and-answer feature) or social features (eg, peer forums) was incorporated into Bolster in a manner consistent with these a priori constraints (eg, providing clinician videos rather than synchronous communication and links to support websites rather than the development of new tools). Third, field trial results may speak primarily to caregiver experiences during a test period rather than the real-world deployment of Bolster. Participants were likely motivated to provide helpful feedback, and this likely affected their frequency of use of the intervention, particularly given the fact that the field trial lasted just 1 week. Interactions with the study team also likely encouraged use. This is particularly the case given the fact that participants received outreach to ensure that the app was functioning. The conditions of a usability field trial may not fully reflect how such an intervention would be deployed under real-world conditions. Finally, analysis of clinical effects was exploratory. The trial was brief and conducted in a small sample of caregivers already connected to treatment.

### Conclusions

The development of Bolster responds to the identified lack of mobile interventions designed for caregivers [40]. Our team’s development of Bolster was grounded in user-centered design and thus seems well suited for future testing to determine whether mHealth can assist caregivers in their critical roles of early identification, treatment facilitation, and ongoing support. These results add to the growing body of literature supporting the use of digital technologies to reduce barriers to treatment and recovery for families affected by psychosis. Bolster seems to respond to specific user-identified interest in more structured, directive, and concrete guidance, as well as support that attends to the emotional experience of caregiving. Future work will examine whether Bolster is an effective tool to support early psychosis caregivers, and our findings here suggest that similar digital approaches to support caregivers of individuals experiencing mental health conditions may have promise. These results demonstrate promise regarding the use of mHealth to support caregivers who—in many settings—lack structured supports to meet their own needs.

## Abbreviations

Brief-COPE: Brief Coping Orientation to Problems Experienced Inventory
CGPS-R: Caregiver Prime Screen–Revised
CSES: Coping Self-Efficacy Scale
DSM-5: Diagnostic and Statistical Manual of Mental Disorders, Fifth Edition
ECI: Experiences of Caregiving Inventory
FQ: Family Questionnaire
GHQ-12: General Health Questionnaire, 12-item version
IPQSR: Illness Perception Questionnaire for Schizophrenia: Relatives’ version
KAST: Knowledge About Schizophrenia Test
mHealth: mobile health
NAMI: National Alliance on Mental Illness
PEPPNET: Psychosis-Risk and Early Psychosis Program Network

## References

[1] Kuipers E (2010). Time for a separate psychosis caregiver service?. J Ment Health.

[2] Anderson KK, Fuhrer R, Malla AK (2013). "There are too many steps before you get to where you need to be": help-seeking by patients with first-episode psychosis. J Ment Health.

[3] Cotton SM, McCann TV, Gleeson JF, Crisp K, Murphy BP, Lubman DI (2013). Coping strategies in carers of young people with a first episode of psychosis. Schizophr Res.

[4] Cheng SC, Backonja U, Buck B, Monroe-DeVita M, Walsh E (2020). Facilitating pathways to care: a qualitative study of the self-reported needs and coping skills of caregivers of young adults diagnosed with early psychosis. J Psychiatr Ment Health Nurs.

[5] Hansen H, Stige SH, Davidson L, Moltu C, Veseth M (2018). How do people experience early intervention services for psychosis? a meta-synthesis. Qual Health Res.

[6] Addington J, van Mastrigt S, Hutchinson J, Addington D (2002). Pathways to care: help seeking behaviour in first episode psychosis. Acta Psychiatr Scand.

[7] Anderson KK, Fuhrer R, Malla AK (2010). The pathways to mental health care of first-episode psychosis patients: a systematic review. Psychol Med.

[8] Lucksted A, Stevenson J, Nossel I, Drapalski A, Piscitelli S, Dixon LB (2018). Family member engagement with early psychosis specialty care. Early Interv Psychiatry.

[9] Stowkowy J, Addington D, Liu L, Hollowell B, Addington J (2012). Predictors of disengagement from treatment in an early psychosis program. Schizophr Res.

[10] Morgan C, Mallett R, Hutchinson G, Bagalkote H, Morgan K, Fearon P, Dazzan P, Boydell J, McKenzie K, Harrison G, Murray R, Jones P, Craig T, Leff J (2005). Pathways to care and ethnicity. 2: source of referral and help-seeking. report from the AESOP study. Br J Psychiatry.

[11] Revier CJ, Reininghaus U, Dutta R, Fearon P, Murray RM, Doody GA, Croudace T, Dazzan P, Heslin M, Onyejiaka A, Kravariti E, Lappin J, Lomas B, Kirkbride JB, Donoghue K, Morgan C, Jones PB (2015). Ten-year outcomes of first-episode psychoses in the MRC ÆSOP-10 study. J Nerv Ment Dis.

[12] Lee G, Barrowclough C, Lobban F (2014). Positive affect in the family environment protects against relapse in first-episode psychosis. Soc Psychiatry Psychiatr Epidemiol.

[13] Wong DT, Tong SF, Daud TI, Aziz SA, Midin M (2019). Factors influencing professional help-seeking behavior during first episode psychosis in schizophrenia: an exploratory study on caregivers' perspective. Front Psychiatry.

[14] Skubby D, Bonfine N, Tracy H, Knepp K, Munetz MR (2015). The help-seeking experiences of parents of children with a first-episode of psychosis. Community Ment Health J.

[15] Onwumere J, Lotey G, Schulz J, James G, Afsharzadegan R, Harvey R, Chu Man L, Kuipers E, Raune D (2017). Burnout in early course psychosis caregivers: the role of illness beliefs and coping styles. Early Interv Psychiatry.

[16] Kuipers E, Onwumere J, Bebbington P (2010). Cognitive model of caregiving in psychosis. Br J Psychiatry.

[17] Pirkis J, Burgess P, Hardy J, Harris M, Slade T, Johnston A (2010). Who cares? a profile of people who care for relatives with a mental disorder. Aust N Z J Psychiatry.

[18] López SR, Gamez D, Mejia Y, Calderon V, Lopez D, Ullman JB, Kopelowicz A (2018). Psychosis literacy among Latinos with first-episode psychosis and their caregivers. Psychiatr Serv.

[19] McCann TV, Lubman DI, Clark E (2011). Responding to stigma: first-time caregivers of young people with first-episode psychosis. Psychiatr Serv.

[20] Fortune DG, Smith JV, Garvey K (2005). Perceptions of psychosis, coping, appraisals, and psychological distress in the relatives of patients with schizophrenia: an exploration using self-regulation theory. Br J Clin Psychol.

[21] Izon E, Berry K, Law H, French P (2018). Expressed emotion (EE) in families of individuals at-risk of developing psychosis: a systematic review. Psychiatry Res.

[22] Leff J, Vaughn C (1981). The role of maintenance therapy and relatives' expressed emotion in relapse of schizophrenia: a two-year follow-up. Br J Psychiatry.

[23] Sadath A, Muralidhar D, Varambally S, Gangadhar BN, Jose JP (2017). Do stress and support matter for caring? the role of perceived stress and social support on expressed emotion of carers of persons with first episode psychosis. Asian J Psychiatr.

[24] Peterson EC, Docherty NM (2004). Expressed emotion, attribution, and control in parents of schizophrenic patients. Psychiatry.

[25] Kuipers E, Bebbington P, Dunn G, Fowler D, Freeman D, Watson P, Hardy A, Garety P (2006). Influence of carer expressed emotion and affect on relapse in non-affective psychosis. Br J Psychiatry.

[26] Finnegan D, Onwumere J, Green C, Freeman D, Garety P, Kuipers E (2014). Negative communication in psychosis: understanding pathways to poorer patient outcomes. J Nerv Ment Dis.

[27] Vasconcelos E Sa D, Wearden A, Hartley S, Emsley R, Barrowclough C (2016). Expressed Emotion and behaviourally controlling interactions in the daily life of dyads experiencing psychosis. Psychiatry Res.

[28] Haidl T, Rosen M, Schultze-Lutter F, Nieman D, Eggers S, Heinimaa M, Juckel G, Heinz A, Morrison A, Linszen D, Salokangas R, Klosterkötter J, Birchwood M, Patterson P, Ruhrmann S (2018). Expressed emotion as a predictor of the first psychotic episode - results of the European Prediction of Psychosis Study. Schizophr Res.

[29] Weintraub MJ, Hall DL, Carbonella JY, Weisman de Mamani A, Hooley JM (2017). Integrity of literature on expressed emotion and relapse in patients with schizophrenia verified by a p-curve analysis. Fam Process.

[30] Rutter M, Brown GW (1966). The reliability and validity of measures of family life and relationships in families containing a psychiatric patient. Soc Psychiatry.

[31] Bebbington P, Kuipers L (1994). The predictive utility of expressed emotion in schizophrenia: an aggregate analysis. Psychol Med.

[32] Alvarez-Jimenez M, Priede A, Hetrick SE, Bendall S, Killackey E, Parker AG, McGorry PD, Gleeson JF (2012). Risk factors for relapse following treatment for first episode psychosis: a systematic review and meta-analysis of longitudinal studies. Schizophr Res.

[33] Buck B, Chander A, Monroe-DeVita M, Cheng SC, Stiles B, Ben-Zeev D (2021). Mobile health for caregivers of young adults with early psychosis: a survey study examining user preferences. Psychiatr Serv.

[34] Vermeulen B, Lauwers H, Spruytte N, Van Audenhove C (2015). P.1.k.038 Experiences of family caregivers for persons with severe mental illness: an international exploration. European Neuropsychopharmacology.

[35] Nahum-Shani I, Smith SN, Spring BJ, Collins LM, Witkiewitz K, Tewari A, Murphy SA (2014). Just-in-time adaptive interventions (JITAIs): an organizing framework for ongoing health behavior support. Methodol Center Tech Rep.

[36] (2018). The Nielsen total audience report: Q1 2018. Nielsen.

[37] Arean PA, Hallgren KA, Jordan JT, Gazzaley A, Atkins DC, Heagerty PJ, Anguera JA (2016). The use and effectiveness of mobile apps for depression: results from a fully remote clinical trial. J Med Internet Res.

[38] Firth J, Torous J, Nicholas J, Carney R, Rosenbaum S, Sarris J (2017). Can smartphone mental health interventions reduce symptoms of anxiety? a meta-analysis of randomized controlled trials. J Affect Disord.

[39] Schlosser DA, Campellone TR, Truong B, Etter K, Vergani S, Komaiko K, Vinogradov S (2018). Efficacy of PRIME, a mobile app intervention designed to improve motivation in young people with schizophrenia. Schizophr Bull.

[40] Onwumere J, Amaral F, Valmaggia LR (2018). Digital technology for caregivers of people with psychosis: systematic review. JMIR Ment Health.

[41] Ben-Zeev D, Kaiser SM, Brenner CJ, Begale M, Duffecy J, Mohr DC (2013). Development and usability testing of FOCUS: a smartphone system for self-management of schizophrenia. Psychiatr Rehabil J.

[42] Ben-Zeev D, Meller S, Snyder J, Attah D, Albright L, Le H, Asafo S, Collins P, Ofori-Atta A (2021). A digital toolkit (M-Healer) to improve care and reduce human rights abuses against people with mental illness in West Africa: user-centered design, development, and usability study. JMIR Ment Health.

[43] Vilardaga R, Rizo J, Zeng E, Kientz JA, Ries R, Otis C, Hernandez K (2018). User-centered design of learn to quit, a smoking cessation smartphone app for people with serious mental illness. JMIR Serious Games.

[44] Schlosser D, Campellone T, Kim D, Truong B, Vergani S, Ward C, Vinogradov S (2016). Feasibility of PRIME: a cognitive neuroscience-informed mobile app intervention to enhance motivated behavior and improve quality of life in recent onset schizophrenia. JMIR Res Protoc.

[45] Kline E, Thompson E, Schimunek C, Reeves G, Bussell K, Pitts SC, Schiffman J (2013). Parent-adolescent agreement on psychosis risk symptoms. Schizophr Res.

[46] Craig TJ, Bromet EJ, Fennig S, Tanenberg-Karant M, Lavelle J, Galambos N (2000). Is there an association between duration of untreated psychosis and 24-month clinical outcome in a first-admission series?. Am J Psychiatry.

[47] Lappin JM, Morgan KD, Morgan C, Dazzan P, Reichenberg A, Zanelli JW, Fearon P, Jones PB, Lloyd T, Tarrant J, Farrant A, Leff J, Murray RM (2007). Duration of untreated psychosis and neuropsychological function in first episode psychosis. Schizophr Res.

[48] Hsieh HF, Shannon SE (2005). Three approaches to qualitative content analysis. Qual Health Res.

[49] Compton MT, Quintero L, Esterberg ML (2007). Assessing knowledge of schizophrenia: development and psychometric properties of a brief, multiple-choice knowledge test for use across various samples. Psychiatry Res.

[50] Lobban F, Barrowclough C, Jones S (2005). Assessing cognitive representations of mental health problems. II. The illness perception questionnaire for schizophrenia: relatives' version. Br J Clin Psychol.

[51] Szmukler GI, Burgess P, Herrman H, Benson A, Colusa S, Bloch S (1996). Caring for relatives with serious mental illness: the development of the Experience of Caregiving Inventory. Soc Psychiatry Psychiatr Epidemiol.

[52] Carver CS (1997). You want to measure coping but your protocol's too long: consider the brief COPE. Int J Behav Med.

[53] Chesney MA, Neilands TB, Chambers DB, Taylor JM, Folkman S (2006). A validity and reliability study of the coping self-efficacy scale. Br J Health Psychol.

[54] Wiedemann G, Rayki O, Feinstein E, Hahlweg K (2002). The Family Questionnaire: development and validation of a new self-report scale for assessing expressed emotion. Psychiatry Res.

[55] Goldberg DP, Blackwell B (1970). Psychiatric illness in general practice. a detailed study using a new method of case identification. Br Med J.

[56] Hardy GE, Shapiro DA, Haynes CE, Rick JE (1999). Validation of the General Health Questionnaire-12: using a sample of employees from England's health care services. Psychological Assessment.

[57] Wang L, Lin W (2011). Wording effects and the dimensionality of the General Health Questionnaire (GHQ-12). Pers Individ Dif.

[58] Ritsner MS, Mar M, Arbitman M, Grinshpoon A (2013). Symptom severity scale of the DSM5 for schizophrenia, and other psychotic disorders: diagnostic validity and clinical feasibility. Psychiatry Res.

[59] American Psychiatric Association (2013). Diagnostic And Statistical Manual Of Mental Disorders, 5th edition.

[60] Gerson R, Davidson L, Booty A, McGlashan T, Malespina D, Pincus HA, Corcoran C (2009). Families' experience with seeking treatment for recent-onset psychosis. Psychiatr Serv.

[61] McCann TV, Lubman DI, Clark E (2011). First-time primary caregivers' experience of caring for young adults with first-episode psychosis. Schizophr Bull.

[62] Shah J, DePietro B, D'Adamo L, Firebaugh M, Laing O, Fowler LA, Smolar L, Sadeh-Sharvit S, Taylor CB, Wilfley DE, Fitzsimmons-Craft EE (2022). Development and usability testing of a chatbot to promote mental health services use among individuals with eating disorders following screening. Int J Eat Disord.

[63] Greer S, Ramo D, Chang Y, Fu M, Moskowitz J, Haritatos J (2019). Use of the chatbot "Vivibot" to deliver positive psychology skills and promote well-being among young people after cancer treatment: randomized controlled feasibility trial. JMIR Mhealth Uhealth.

[64] Fitzpatrick KK, Darcy A, Vierhile M (2017). Delivering cognitive behavior therapy to young adults with symptoms of depression and anxiety using a fully automated conversational agent (Woebot): a randomized controlled trial. JMIR Ment Health.

[65] Cascella M, Montomoli J, Bellini V, Bignami E (2023). Evaluating the feasibility of ChatGPT in healthcare: an analysis of multiple clinical and research scenarios. J Med Syst.

[66] Inkster B, Sarda S, Subramanian V (2018). An empathy-driven, conversational artificial intelligence agent (Wysa) for digital mental well-being: real-world data evaluation mixed-methods study. JMIR Mhealth Uhealth.

[67] Evans-Lacko S, Hahn JS, Peter LJ, Schomerus G (2022). The impact of digital interventions on help-seeking behaviour for mental health problems: a systematic literature review. Curr Opin Psychiatry.

[68] McFarlane WR, Dixon L, Lukens E, Lucksted A (2003). Family psychoeducation and schizophrenia: a review of the literature. J Marital Fam Ther.

[69] Lucksted A, McFarlane W, Downing D, Dixon L (2012). Recent developments in family psychoeducation as an evidence-based practice. J Marital Fam Ther.

[70] Rotondi AJ, Anderson CM, Haas GL, Eack SM, Spring MB, Ganguli R, Newhill C, Rosenstock J (2010). Web-based psychoeducational intervention for persons with schizophrenia and their supporters: one-year outcomes. Psychiatr Serv.

[71] Kopelovich SL, Stiles B, Monroe-DeVita M, Hardy K, Hallgren K, Turkington D (2021). Psychosis REACH: effects of a brief CBT-informed training for family and caregivers of individuals with psychosis. Psychiatr Serv.

